# Effects of biological and non-biological immunomodulatory therapies on the immunogenicity of vaccines in patients with rheumatic diseases

**DOI:** 10.1186/s13075-014-0506-0

**Published:** 2014-12-23

**Authors:** Zsuzsanna H McMahan, Clifton O Bingham III

**Affiliations:** Department of Medicine, Division of Rheumatology, Johns Hopkins University, 5200 Eastern Avenue, Mason F Lord Center Tower, Room 4100, Baltimore, MD 21224 USA

## Abstract

Vaccinations are administered to patients to induce a protective immune response, resulting in immunological memory. Preventing infection through the use of vaccines is particularly important in immunocompromised and immunosuppressed individuals given their increased frequency and severity of infections relative to healthy individuals. Recent surveys show that the vaccination rate is still alarmingly low in patients with rheumatic disease. In this review we briefly discuss the different types of vaccines and then critically examine evidence related to vaccination efficacy in patients with autoimmune disease and the effects of immunomodulatory therapy, with an aim to provide guidance and optimize the administration of vaccines in such individuals.

## Introduction

Immunizations are effective in inducing and/or enhancing protective immunity, and may be particularly important in the immunocompromised. Autoimmunity often imparts an increased risk for infection with additional medication-related risks, and common infections have higher morbidity and mortality. Optimizing care for patients with autoimmune disease requires treatment of the underlying disease and minimizing infection-related comorbidity, with vaccination as an important component.

Treatments for rheumatic diseases have greatly expanded in the last 15 years, encompassing biological and nonbiological pathway inhibitors, all associated with infections, and with consequent interest in mitigating this risk. Patients taking immunomodulators have suboptimal rates of immunizations, due in part to concerns over vaccine-associated adverse events, possible activation of the underlying autoimmune process, and questions of vaccine efficacy [1,2]. New guidelines and recommendations provide conflicting information and/or inadequately address specific rheumatologic concerns. Proportions of rheumatologic patients vaccinated for influenza are 40% or less, and in US Medicare recipients, only 33% of rheumatoid arthritis (RA) and psoriatic arthritis patients received a pneumococcal vaccine over a 5 year period [1].

Our goals are to critically examine evidence regarding immunization efficacy in autoimmune diseases and with the array of immunomodulatory agents used in the management of these patients. This review will provide data to inform decisions to optimize vaccine efficacy, avoid adverse events, and reduce infectious risk.

## Methods

We conducted this review to evaluate immunization efficacy in rheumatic conditions and with different immunomodulators, including both biological and non-biological agents. An initial exhaustive literature search used PubMed with terms listed in Table 1. Relevant abstracts from 2007 to 2013, American College of Rheumatology (ACR) and European League Against Rheumatism (EULAR) meetings were included as well as guidelines and recommendations from immunological, infectious diseases, and rheumatologic societies, and the Centers for Disease Control (CDC), and descriptions of studies and results from ClinicalTrials.gov. Additional articles were obtained from reference lists and authors’ personal collections. An initial search resulted in 7,226 articles, which were screened and selected for clinical studies and reviews evaluating responses to vaccines in patients with autoimmune diseases and transplants receiving immunomodulatory therapy. After selection was complete, 147 papers were reviewed in depth. Among studies there was significant heterogeneity; in some studies immune responses were reported for patients receiving different medications, and in some participants were taking multiple agents. We extracted data from these articles by drug if possible for presentation, and have presented summaries for each of the drugs and/or classes using the most salient examples and references. Given the paucity of published information for many immunomodulatory therapies currently being used, we have also included abstract data from ACR and EULAR meetings to provide clinicians with additional data to inform clinical decision-making.

**Table 1:** **Search terms**

Search terms
immunization, vaccines, vaccination, systemic lupus erythematosus, vasculitis, rheumatoid arthritis, limited and diffuse scleroderma, systemic sclerosis, myositis, juvenile rheumatoid arthritis, discoid lupus erythematosus, autoimmune diseases, transplants, pediatric, hydroxycorticosteroids, glucocorticoids, cyclosporine, sirolimus, tacrolimus, mycophenolate mofetil, azathioprine, 6-mercaptopurine, methotrexate, hydroxychloroquine, sulfasalazine, leflunomide, TNFR-Fc fusion protein, etanercept, abatacept, rituximab, tocilizumab, infliximab, adalimumab, CDP870, certolizumab, and golimumab, limited to articles after 1980

## Immunizations: general overview

Vaccines vary in their components, including recombinant or purified protein antigens, live attenuated or killed organisms, carbohydrate and polysaccharide antigens, and conjugates (Table 2). Each mechanism activates different immunological pathways with efficacy and safety implications and may invoke a neoantigen or booster response.

**Table 2:** **Types of vaccines and examples**

Carbohydrate/polysaccharide antigens	Protein antigen: recombinant/inactivated/conjugated**	Live/attenuated organisms
Pneumococcal polysaccharide (PPSV-23, for example, Pneumovax®)	Tetanus, diphtheria, acellular pertussis (TD/DT, TDAP, DTAP)	Varicella (VZV, Varivax®, Varilrix®)
Meningococcal polysaccharide (MPSV-4)	Hepatitis A, hepatitis B	Shingles, zoster (for example, Zostavax®)
Typhoid polysaccharide (Vi injection)	Seasonal influenza A/B injection	Intranasal influenza (for example, Flu-mist®)
	Pandemic influenza (H1N1) injection	
	Human papilloma virus	Measles, mumps, rubella
	Anthrax (acellular)	Yellow fever
	Inactivated polio (IPV, Salk, IM/SQ)	Oral polio (OPV)
	Oral cholera (killed cells)	Typhoid (Ty21a oral)
	Pneumococcal conjugate** (PCV-7, PCV-13, for example, Prevnar®)	Vaccinia (smallpox)
	Meningococcal conjugate** (MCV-4, <55 years old)	Bacillus Calmette-Guérin
	Haemophilus influenza type B protein polysaccharide conjugate** (HiB, PRP)	Rotavirus
		Anthrax (live spore)
		Smallpox

Vaccinations may vary in terms of their constituents from country to country (for example, Japanese encephalitis virus, rabies, anthrax) and over time as new vaccines are developed. Providers are advised to consult product inserts of specific vaccines to confirm constituents before use. DTAP, diphtheria, tetanus, and pertussis; HiB, Haemophilus influenza type B; IM/SQ, intramuscular/subcutaneous; IPV, inactivated polio virus; MCV-4, quadrivalent meningococcal conjugate; MPSV-4, quadravalent meningococcal polysaccharide vaccine; OPV, oral polio virus; PCV, pneumococcal conjugate vaccine; PPSV, pneumococcal polysaccharide vaccine; PRP, polyribosylribitol phosphate; TD/DT, Tetanus Diphtheria/Diphtheria Tetanus; TDAP, Tetanus diphtheria acellular pertussis; Vi, Vi capsular polysaccharide; VZV, varicella zoster vaccine. **conjugated vaccines.

Live organism vaccines, typically viruses with attenuated virulence but also live bacteria, generally induce high titer and long-lasting immune responses (Table 3). Most are viruses (for example, zoster, varicella, yellow fever, measles/mumps/rubella (MMR), intranasal influenza, oral polio, and rotavirus) but some are live bacteria (for example, oral typhoid, Bacillus Calmette-Guérin). Their recognition is of paramount importance for immunosuppressed patients because they may induce illness even from an attenuated strain [3]; thus, live vaccines are not recommended for patients on many disease-modifying anti-rheumatic drugs (DMARDs) and immunomodulators.

**Table 3:** **Summary of data for vaccine efficacy and safety with immunomodulatory therapies**

**Drug**	**Protein vaccines**	**Carbohydrate vaccines**	**DTH/cellular immunity**	**Neoantigen**	**Live virus**
**Non-biologic immunomodulators**					
Corticosteroids	--/↓	--	ND	ND	Zoster OK with CCS <20 mg/day
Methotrexate	↓↓	↓	--	--	Zoster OK with MTX <0.4 mg/kg/week
Anti-malarials	--	--	ND	ND	Probably safe, possible ↓ response
Sulfasalazine	--/↓	ND	ND	ND	Probably safe, not formally studied
Leflunomide	--	ND	ND	ND	ND
Azathioprine	--	--/↓	ND	ND	Zoster OK <3 mg/kg/day
Mycophenolate	↓↓	↓↓	↓	↓	Avoid
Calcineurin Inhibitors	--/↓	ND	↓	ND	Avoid
**Biologicals and targeted immunomodulators**					
TNF inhibitors	--/↓	--/↓	--	ND	Avoid
Abatacept (CTLA4-Ig)	↓	↓	ND	↓	Avoid
Rituximab (anti-CD20)	--/↓	↓↓	↓	↓↓	Avoid
Tocilizumab (anti-IL6)	--	--	ND	ND	Avoid
Ustekinumab (anti-IL-12/23)	--	--	ND	ND	Avoid
IL**-**1 inhibitors (anakinra, Rilonacept, canakinumab)	ND	ND	ND	ND	Avoid
Belimumab (anti-BLyS)	ND	ND	ND	ND	Avoid
Tofacitinib (Jak1/3)	--/↓	↓	ND	ND	Avoid

↓ decreased, ↓↓ markedly decreased, -- no effect. BLyS, B lymphocyte stimulator; CCS, corticosteroids; DTH, delayed type hypersensitivity; MTX, methotrexate; ND, not determined; TNF, tumor necrosis factor.

There is a paucity of data available, but the CDC has provided recommendations for the immunosuppressed with some vaccines and particular drugs; however, categorization of immunosuppression is quite broad and includes congenital immunodeficiencies, HIV infection, chemotherapy-induced marrow ablation, transplant-related immunosuppression, and rheumatic disease patients receiving immunomodulators. The attenuated live varicella zoster vaccine is recommended for adults aged over 60 years, regardless of prior chicken pox infection, immunization, serology, or shingles history and may be used in patients aged 50 to 59 years with potentially poor tolerance to zoster infection or post-herpetic neuralgia. CDC guidance has been published for zoster vaccine use in the immunosuppressed [4]. The vaccine may be given to patients with decreased humoral immunity (that is, isolated immunoglobulin (Ig) deficiency), patients receiving low to moderate dose steroids (<20 mg/day prednisone), and patients on >20 mg prednisone for ≥2 weeks after 1 month of discontinuation. The zoster vaccine can be used with doses of methotrexate (MTX), azathioprine (AZA), and 6-mercaptopurine typically used in autoimmune disease treatment.

Concerns remain regarding live vaccine safety with other biological therapies and immunosuppressants. Although recent reports using managed care and Medicare/Medicaid databases suggest that patients receiving tumor necrosis factor (TNF) inhibitors may be able to receive the zoster vaccine without adverse effect [5,6], further prospective studies are needed. In juvenile rheumatic disease patients on MTX and corticosteroids the live varicella vaccine did not cause any overt infections [7]. While there is a concern for potential conversion to a virulent form of varicella in the immunocompromised, effective antiviral drugs such as acyclovir and valacyclovir are available to treat acute infections should they arise. In patients receiving the zoster vaccine, these antivirals should be discontinued before immunization and several weeks thereafter to maximize response. MMR vaccines have also been administered to children with pediatric rheumatic diseases receiving immunodulators. In one study, there were no cases of viral activation or illness with MMR in children with juvenile idiopathic arthritis (JIA) taking MTX or etanercept [8]. An important caveat for zoster and MMR vaccine studies is that these largely represented recall or booster responses of preexisting immunological memory, which may have attenuated the risk of virulent conversion. The yellow fever vaccine is also a live virus and required for travel to many regions in the world, thus representing a neoantigen for most. Although the live yellow fever vaccine was given without sequelae to 17 Brazilian RA patients receiving infliximab, this was as part of a re-immunization effort rather than a neoantigen response; these results cannot be extrapolated to immunocompromised individuals receiving yellow fever vaccine for the first time [9]. There are limited data concerning the risk to immunosuppressed individuals of acquiring infection from a family member or close contact who receives a live virus vaccine [10]. In studies of live attenuated intranasal influenza vaccine administration in pediatric cancer [11], viral shedding was limited to <7 to 10 days, but the vaccine is not recommended by the CDC in the immunocompromised or adults aged over 50 years [12]. Household contacts of immunosuppressed patients without immunity may also receive live virus vaccines, as transmission of attenuated vaccine strain virus is rare, but caution should still be exercised. Recently published recommendations concerning viral transmission risk to patients with immunodeficiencies from family contacts receiving vaccines noted that these events were possible but rare [10].

Recombinant protein antigen vaccines and killed virus vaccines are also used but may have decreased immunogenicity (relative to live vaccines) and require multiple doses. Polysaccharide or carbohydrate antigen vaccines were developed for some encapsulated organisms, including pneumococcus (23-valent pneumococcal polysaccharide vaccine (PPSV-23), Pneumovax®), and meningococcus (quadravalent meningococcal polysaccharide vaccine-4). Polysaccharide immunization responses largely reflect T-cell-independent mechanisms. Protein-conjugate vaccines, such as 7- and 13-valent pneumococcal conjugate (PCV-7, PCV-13 or Prevnar®), meningococcal conjugate (MCV-4), and conjugated Haemophilus influenza type B (HiB) attach polysaccharide antigens to a carrier protein to enhance response. The CDC Advisory Committee on Immunization Practices now recommends PCV-13 to all immunocompromised adults aged over 19 years, followed ≥8 weeks later by PPSV-23 to induce optimal memory, with subsequent PPSV-23 every 5 years [12,13]. In patients who received prior PPSV-23, at least 1 year is recommended before giving PCV-13.

## Measuring vaccine responses

One difficulty in evaluating various vaccine studies is a lack of standardized reporting. Although vaccines elicit cellular and humoral immunity, efficacy is primarily measured by antibody titers. Four general parameters are used to measure vaccine response: 1) geometric mean titers (GMTs), 2) seroprotection rate, 3) seroconversion rate, and 4) seroconversion factor. A four-fold rise in GMT is one measure of vaccine efficacy, though some studies report two-fold increases. The seroprotection rate represents the percentage of recipients with an antibody titer at which the probability of protection is assumed to be 50%, but protective levels are poorly standardized and may vary among studies and between vaccines, and indeed between individuals. Seroconversion rates describe the percentage of recipients with a two- to four-fold or more increase in post-vaccination titers. The seroconversion factor is defined as the post-vaccination titer divided by the pre-vaccination titer. Some studies have shown adequate seroprotection but without expected increases in antibody titers, attributed to cellular immunity. Other studies have only reported increases in titers for patients without baseline levels of protective titers (rather than all patients receiving the vaccine), leading to difficulties in comparing study results. For the majority of vaccines, the protective correlates, especially cellular immune correlates and nasal and/or serum IgA, are poorly defined. Hence, in a majority of cases, only serum antibody-mediated assessments serve as correlates due to the complexity of assessment of cell-mediated immune responses.

Antibody responses reported as endpoints in most studies reflect a defined point in time (typically 4 weeks after immunization), but do not necessarily reflect titer maintenance. Although mounting an early response is assumed to result in development of immunological memory, few studies have evaluated titer durability over time or the efficacy of boosting in immunocompromised patients. Strategies to increase vaccine responses include addition of adjuvant, booster vaccinations, increasing antigen dose, and intradermal administration.

## Results

**Figure 1:**
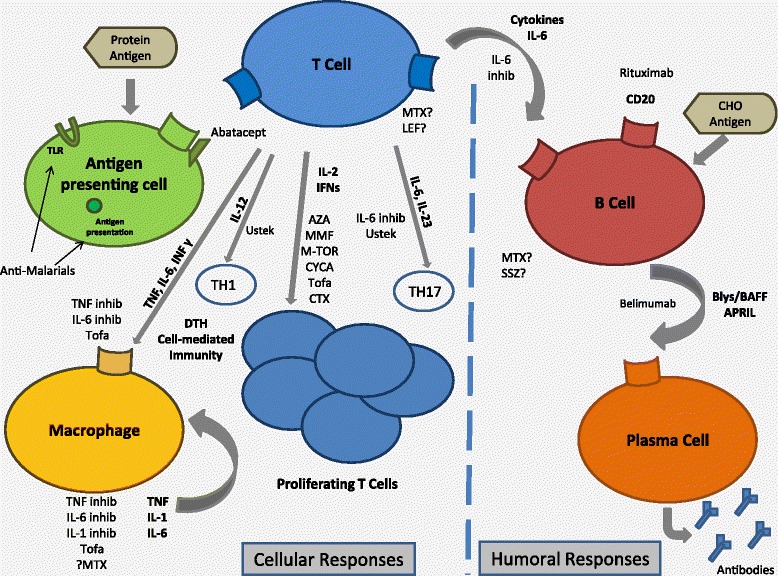
**Immunomodulatory therapies.** AZA, azathioprine; BAFF, B-cell activating factor; Blys, B lymphocyte stimulator; CHO, carbohydrate; CTX, cyclophosphamide; CYCA, cyclosporine A; DTH, delayed type hypersensitivity; INF, interferon; inhib, inhibitor; LEF, leflunomide; MMF, mycophenolate mofetil; M-TOR, mammalian target of rapamycin; MTX, methotrexate; SSZ, sulfasalazine; TLR, toll-like receptor; Tofa, tofacitinib; Ustek, ustekinumab.

Rheumatic diseases are managed with a variety of immunomodulators, ranging from corticosteroids to non-biological and biologic DMARDS and immunosuppressives (Figure 1).

### Non-biological agents

#### Corticosteroids

Immune responses of autoimmune disease patients taking steroids have been well studied [14]. Even at low doses overall infection and opportunistic infections are increased [15,16]. Studies evaluated systemic lupus erythematosus (SLE), JIA, polyangiitis with granulomatosis, scleroderma, RA, and renal transplants, with influenza, tetanus, PPSV-23, HiB, and hepatitis B virus (HBV) vaccines. In most, glucocorticoids impaired vaccine response, and decreased cellular responses to diphtheria and tetanus were reported in transplant patients receiving steroids.

#### Methotrexate

Many studies evaluated responses to vaccines with MTX, most in RA, but also JIA, psoriatic arthritis, and SLE [8,14,17–19]. Vaccines included PPSV-23, PCV-7, HBV, influenza, MMR, the neoantigen keyhole limpet hemocyanin (KLH), and delayed type hypersensitivity (DTH) reactions. In 17 RA patients on MTX (mean dose 11.6 mg/week) responses to recombinant HBV protein vaccine were seen in 68%, and use of MTX was not associated with lack of response [14]. In two studies, RA patients on MTX responded similarly to healthy controls for influenza [19], with GMTs significantly increased against each strain; however, the percentage of responders was lower in RA. Pneumococcal responses were also evaluated with MTX, using PPSV-23 or PCV-7 [20]. In a direct comparison, responses to PPSV-23 were similar to those to PCV-7; but MTX has emerged as a predictor of poor response, with a greater effect than TNF inhibitors [20,21].

Bingham and colleagues reported immune responses in 20 RA patients on MTX monotherapy to tetanus toxoid (TT), PPSV-23, KLH, and DTH. TT responses were seen in only 42.3% on MTX, lower than that expected for ‘normal’ controls (>70 to 80%) [18]. Responses to PPSV-23 ranged from 29% to 61% for individual serotypes, but 79% responded to ≥3 serotypes. Ninety-three percent treated with MTX had detectable anti-KLH IgG neoantigen responses, and most (70%) maintained positive DTH response. In a more recent study, 23 RA patients on MTX monotherapy had similar reductions in TT (39% responders) and PPSV-23 responses (70.8% with 6 serotypes) [22].

In 10 JIA patients on MTX who received MMR booster vaccinations, those treated with MTX alone had reduced humoral responses, and while there was no reduction in titer with MTX plus TNF inhibitors, markers of cell-mediated immunity were reduced [8]. Seroprotection rates and GMT for mumps, rubella, diphtheria, and tetanus were recently reported to be lower in JIA than controls, but unrelated to MTX exposure [23].

In summary, MTX may reduce response in patients receiving some protein antigens (influenza and TT) but retention of others (for example, KLH). Reductions in polysaccharide and conjugated pneumococcal responses are reported, but most individuals mount responses to multiple serotypes. The safety of live virus booster vaccines was demonstrated for MMR with MTX, and zoster vaccines may also be administered to these patients.

#### Other non-biological DMARDs

Immunization efficacy was studied with chloroquine and hydroxychloroquine (HCQ) in SLE and RA, and healthy individuals for malaria prophylaxis [24]. Responses to yellow fever, oral polio, oral cholera and typhoid vaccines, PPSV-23, human papilloma virus (HPV), and influenza vaccines were analyzed. Little or no impairment in responses were reported with anti-malarials [25]. Importantly, no illness or activation of live attenuated yellow fever vaccine occurred. One study evaluated immunogenicity and safety of HPV vaccine in 50 women with SLE and 50 healthy controls; among the 66% of SLE patients on HCQ, there was no significant reduction in seroconversion rates [26].

Immunization efficacy with sulfasalazine (SSZ) has also been studied [27,28]. In one study, responses to TT or inactivated influenza were evaluated in 23 healthy subjects receiving SSZ or placebo, demonstrating a significant reduction in IgG and IgA anti-tetanus-producing cells [27]. In a contrasting study, SSZ had no effect on immunization responses to adjuvented pandemic influenza vaccine [25]. In seven JIA patients on SSZ only one was a ‘low responder,’ to MCV-4 [29].

Vaccination responses with leflunomide have not been well-studied. Ribeiro and colleagues [30] evaluated immune responses to the trivalent influenza vaccine in RA. Among 340 patients on combinations of DMARDs (42.9% on leflunomide) compared with 230 healthy controls, leflunomide use was not associated with decreased immune response.

#### Azathioprine

A considerable amount of data is available for AZA in patients with SLE, inflammatory arthritis, polyangiitis with granulomatosis, and renal transplant using different vaccines (influenza, injectable typhoid, PPSV, TT, and HiB) [31–33]. Studies of influenza responses are conflicting between reports, with some showing no reductions in responses [32] and others demonstrating decreased seroconversion, reduction in GMTs, and fewer four-fold titer increases with AZA [31,32]. Responses to other vaccines, including TT, PPSV-23, HBV, and the HPV vaccine were not, however, significantly decreased with AZA. Across these studies variable endpoints were used to define a response [26].

#### Mycophenolate

Influenza vaccine responses, seroprotection rates, and seroconversion rates were reduced with mycophenolate (MMF) compared with AZA in transplant recipients [34]. In another transplant study that evaluated cellular, humoral, and recall responses to PPSV and TT vaccines, patients on MMF were unable to mount a primary humoral or recall response to TT and PPSV-23, and cellular responses to TT were also reduced [35]. With the HPV vaccine in SLE, those on MMF had reduced seroconversion rates for two genotypes compared with patients receiving other immunodulators (HCQ, AZA, and calcineurin inhibitors), and titers were inversely correlated with MMF dose [26]. There are limited data on the more potent immunosuppressant, cyclophosphamide, in rheumatic diseases. The response to PPSV-23 in two patients with systemic sclerosis on cyclophosphamide was severely impaired [36].

#### Cyclosporine and calcineurin inhibitors

The effects of cyclosporine (CycA) on immune responses have been studied in transplant recipients with influenza, KLH, TT, and HBV [37,38]. In these studies, controls were typically patients receiving other immunomodulators (for example, AZA, MMF, sirolimus), and usually steroids [35,39]. In one study, CycA-treated patients had a lower immune response against influenza A than AZA-treated patients, and boosters were not effective. Another group reported significantly decreased responses to influenza with CycA compared with sirolimus in lung transplants. In CycA-treated chronic uveitis patients, significantly decreased DTH was seen, but without differences in responses to KLH or TT [37].

### Biologics

#### Tumor necrosis factor inhibitors

Several studies, mostly in RA, have evaluated immunizations with TNF inhibitors [17,21,39–42]. Influenza and pneumococcal responses were the focus of most, but MMR was studied in JIA. Overall, vaccination with TNF monotherapy was effective, but a combination of MTX with TNF inhibitors showed significantly decreased responses [20,21]. In two JIA studies with the conjugate pneumococcal vaccine (PCV-7) or MMR re-vaccination, MTX and etanercept did not decrease responses. One small study demonstrated that there was no reduction in DTH responses with etanercept [43]. Although live virus vaccines are not recommended in patients receiving biologics, the incidence of herpes zoster in patients receiving TNF inhibitors was not increased compared with non-biological DMARDs [6]. Although a small study reported that booster live attenuated yellow fever vaccines did not cause illness, these were recall responses and not first exposure to the vaccine [9]. Overall, the available data would support that vaccination with non-live viruses in patients receiving TNF inhibitors may have slight reductions in response, but these are likely protective. While some data suggest that certain live virus vaccines eliciting booster or recall responses may be safe in patients receiving TNF inhibitors (for example, zoster, MMR), extrapolation to live organism neoantigen immunizations cannot be assumed, and additional studies are necessary.

#### Abatacept

Immunization responses with abatacept, which blocks T-cell activation via co-stimulatory pathways, were studied in RA, psoriasis, and healthy controls. In healthy individuals, abatacept did not inhibit responses to TT or PPSV-23 [44]. Patients with RA on abatacept vaccinated with the conjugated PCV-7 had a diminished response [45], with only 48% mounting a response to ≥3 serotypes. Psoriasis patients on abatacept showed decreased responses to both the bacteriophage PhiX and KLH neoantigens [46]. A recent open-label study reported the efficacy of PPSV-23 and seasonal influenza vaccines in RA patients taking subcutaneous abatacept plus background DMARDS. In those without protective titers at baseline, 73.9% mounted a response to PPSV-23 (defined as a two-fold increase in three out of five antigens), with 83.9% developing protective titers. Only 61.3% had a response to influenza vaccine (four-fold increase in two out of three antigens), but 82.1% had protective titers [47].

#### Rituximab

By inhibiting B cells, rituximab (RTX) may affect humoral immunity as evidenced by total IgM and IgG slowly declining with repeated administration. In rheumatic disease patients, a number of studies have evaluated vaccine responses with RTX with influenza TT, PPSV-23, DTH, and KLH, mostly in RA [19,48]. In a large study of RA patients on RTX and MTX, immunizations were given after a first course of RTX during the period of B-cell depletion. Responses to TT and DTH were preserved at 24 weeks compared with MTX alone, but neoantigen responses (KLH) and responses to PPSV-23 were significantly reduced [18]. In other RA studies, patients on RTX had significantly diminished responses to influenza vaccine, with one group showing better responses as a booster response than as a neoantigen [19,48]. Although humoral responses were reduced, T-cell responses to vaccine antigens were similar with RTX compared with other DMARDs [49]. Because patients treated with RTX may have potentially fatal reactivation of latent viral infections, live virus vaccines should be strictly avoided. Current vaccine studies of RTX are limited to treatment with a single course of therapy. Whether responses would be impaired further with subsequent courses requires additional study.

#### Belimumab

A recent study reported responses to PPSV-23, influenza, and TT vaccines in SLE patients receiving belimumab directed against the cytokine B lymphocyte stimulator involved in plasma cell differentiation [50]. Belimumab had no significant effect on the maintenance of antibody titers in previously vaccinated patients, but there were no controls, and vaccine exposure was not standardized.

#### Tocilizumab

Interleukin (IL)-6 has pleiotropic activity, including effects on B-cell and T-cell differentiation and development. In a small open-label study protective antibody titers to influenza were reached in over 70% of tocilizumab (TCZ)-treated RA patients [51]. A controlled study (TCZ plus MTX versus MTX) was conducted in RA patients who received TCZ (8 mg/kg), with TT and PPSV-23 given after 3 weeks and responses measured at 8 weeks after one additional TCZ infusion. Numerically more patients responded to PPSV-23 based on a two-fold increase in titer in ≥6 serotypes with MTX versus TCZ plus MTX (70.8% versus 60%), but confidence intervals were overlapping. Responses to TT were seen in only 39% of patients receiving MTX alone, but not further attenuated with TCZ plus MTX (42%) [52]. A limitation of this study was exposure to only two TCZ infusions at the time of immunization. To date, no information is available using other IL-6 inhibitors in development.

#### Ustekinumab

There has been increasing appreciation of the role of the IL-12/23 and IL-17 pathways in several rheumatic diseases. Ustekinumab (UST), which binds and inhibits signaling by both IL-12 and IL-23, has been approved for the treatment of psoriatic arthritis. Responses to TT and PPSV-23 were examined in patients with psoriasis receiving prolonged treatment with UST (n = 60) and compared with a control group of psoriasis patients not taking systemic therapy (n = 56). There was no demonstrable impairment in responses to PPSV-23; >2-fold increases were seen for ≥7 of 14 pneumococcal serotypes in 96.6% of UST-treated patients and 92.6% of controls. Similarly, there was no decrement in responses to TT (≥4-fold increase) in patients following vaccination, with 84.7% of UST-treated and 77.8% of controls having responses. Cellular responses to TT were also examined and showed no difference between groups [53].

#### Interleukin-1 inhibitors

There is limited information on immunization responses in patients receiving anakinra, an IL-1 receptor antagonist approved for RA, the IL-1R-Accessory Protein fusion protein rilonacept, or the anti-IL1-β antibody canakinumab. The effects of anakinra on vaccine antibody responses in patients with RA were studied, but these results have never been presented.

#### Tofacitinib

The most recently approved agent in the US for the treatment of RA is the Janus kinase (Jak) inhibitor tofacitinib, which preferentially blocks Jaks 1 and 3, with additional Jak inhibitors in development. While not ‘biologic’ in terms of its molecular structure, it inhibits downstream activities of multiple cytokine and growth factors, including IL-6 and the T-cell cytokines IL-2 and interferons. To date, only abstract data have been presented concerning immunization efficacy with tofacitinib. In the first study, PPSV-23 and influenza vaccines were administered to patients taking long-term tofacitinib, some stopping tofacitinib 2 weeks before immunization and others without tofacitinib interruption. There was no significant difference in response to PPSV-23 (>2-fold increase in 6 of 12 antigens; 75% versus 84.6%) or influenza (>4-fold increase in 2 of 3 antigens; 66.3% versus 61.8%) between continuous exposure and 2-week tofacitinib withdrawal, but titers were higher in those taken off tofacitinib before immunization [54]. A larger study was conducted in RA patients on 10 mg bid of tofacitinib alone or placebo stratified for MTX use using influenza and PPSV-23 vaccines, with response measured at 35 days. Similar proportions of patients had responses to influenza (≥4-fold increase in 2 of 3 antigens) in tofacitinib versus placebo (56.9% versus 62.2%) with some reductions seen in those on MTX. With PPSV-23, only 45.1% of those on tofacitinib had a response (≥2-fold increase in 6 of 12 antigens) compared with 68.4% on placebo, with the differences most notable in those on MTX versus no MTX as co-therapy [54,55]. As with other immunomodulators, live virus vaccines in patients treated with tofacitinib should be avoided.

## Conclusion

Immunizations remain an important component of risk mitigation for patients receiving immunomodulatory therapies, but there are important caveats to consider for individual patients and with specific drugs in terms of efficacy and potentially with safety. Vaccines remain immunogenic, although responses may be somewhat attenuated with certain drugs. The magnitude of the immune response to vaccines in RA depends on 1) pre-existing memory at the T- and B-cell level as the antigen and co-stimulatory threshold requirements are lower for memory populations, 2) the nature of the immunosuppressant (B-cell depleting versus others), 3) the timing of vaccination, and 4) whether or not the vaccine is adjuvented. The optimal time for administration to achieve optimal titers and memory is ideally before the initiation of immunomodulators, though this may not always be possible. Most routine vaccinations should be administered according to current guidelines to all patients with rheumatic diseases taking immunosuppressive medications.

Live vaccines should be avoided in patients taking most immunosuppressive and immunomodulatory therapies, ideally administered prior to DMARD initiation. The zoster vaccination may be safe in patients receiving MTX, AZA, or moderately dosed prednisone. While MMR has been administered as a booster to children taking MTX and TNF inhibitors without reported sequelae, this has not been formally studied. There are limited data regarding the safety of other live virus vaccines (for example, intranasal influenza, yellow fever) in rheumatic disease patients taking DMARDs or biologics. While possible, live viral shedding from household members or close contacts to rheumatic disease patients taking immunosuppressive therapies has not been well documented. If live virus vaccines are required in patients on immunosuppressives, there is no evidence base upon which to provide guidance for how long a patient should be off a particular agent before receipt of the vaccine; this would need to factor in both the pharmacokinetics as well as the pharmacodynamics of individual compounds. The potential of utilizing a combination of human monoclonal antibodies against infectious agents may be a focus of future applications and could expand vaccine applications.

Recommendations for vaccination in patients with rheumatic diseases have been published by EULAR and ACR based on available evidence and expert consensus. Since their publication, a number of new drugs have been approved, and additional data have been presented as abstracts and manuscripts for already approved drugs discussed in this paper, and additional vaccine recommendations have been issued regarding immunizations in general and in the immunocompromised. These include recommendations for pertussis inclusion with tetanus boosters in all individuals, given the resurgence of whooping cough and waning immunity in adults and recommendations for the conjugated PCV-13 in immunosuppressed adults followed by PPSV-23.

More controlled studies are needed to evaluate immunization efficacy with existing agents and as part of the evaluation of new drugs. To interpret such data, controls with the disease under study taking an alternative standard regimen (for example, MTX in RA studies) and healthy controls are needed. The current lack of standardized reporting of results from immunization studies makes cross-study comparisons difficult. Additional evaluation of humoral and cellular immune responses following prolonged periods of immunomodulatory use is needed. Finally, studying booster immunizations for suboptimal responders and to maintain titers in those on immunosuppressants, and the optimal timing of vaccine administration are also areas that merit further investigation.

## Abbreviations

ACR: American College of Rheumatology
AZA: Azathioprine
CDC: Centers for Disease Control
CycA: Cyclosporine
DMARD: Disease-modifying anti-rheumatic drug
DTH: Delayed type hypersensitivity
EULAR: European League Against Rheumatism
GMT: Geometric mean titer
HBV: Hepatitis B virus
HCQ: Hydroxychloroquine
HiB: Haemophilus influenza type B
HPV: Human papilloma virus
Ig: Immunoglobulin
IL: Interleukin
Jak: Janus kinase
JIA: Juvenile idiopathic arthritis
KLH: Keyhole limpet hemocyanin
MCV: Meningococcal conjugate vaccine
MMF: Mycophenolate
MMR: Measles/mumps/rubella
MTX: Methotrexate
PCV: Pneumococcal conjugate vaccine
PPSV: Pneumococcal polysaccharide vaccine
RA: Rheumatoid arthritis
RTX: Rituximab
SLE: Systemic lupus erythematosus
SSZ: Sulfasalazine
TCZ: Tocilizumab
TNF: Tumor necrosis factor
TT: Tetanus toxoid
UST: Ustekinumab
